# Exosomal lipids induce human pancreatic tumoral MiaPaCa-2 cells resistance through the CXCR4-SDF-1α signaling axis

**DOI:** 10.18632/oncoscience.96

**Published:** 2014-11-11

**Authors:** Sadia Beloribi-Djefaflia, Carole Siret, Dominique Lombardo

**Affiliations:** ^1^ Aix-Marseille Université, CRO2, INSERM, UMR 911, Marseille cedex 5, France

**Keywords:** Exosomes, pancreatic neoplasm, cancer cells, lipid rafts, CXCR4-SDF-1α survival axis

## Abstract

We previously reported that exosomes secreted by human pancreatic tumor cells induce cell death through the inhibition of the Notch-1 survival pathway (Ristorcelli *et al*., 2009). We demonstrated that exosomal lipids evoked apoptosis of human pancreatic cancer SOJ-6 cells. Based on the lipid composition of efficient exosomes we designed Synthetic Exosome-Like Nanoparticles (SELN) in which the ratio ordered lipids versus disordered lipids was equal to 6.0 (SELN6.0). These SELN decreased SOJ-6 cells survival by inhibiting the Notch-1 pathway. However MiaPaCa-2 cells were resistant to exosomes (Ristorcelli *et al*., 2008) and to SELN6.0 (Beloribi *et al*.,2012). In this paper we aimed at deciphering the reason(s) of this resistance. We observed, in presence of SELN6.0, that the expression of the Notch IntraCytoplasmic Domain (NICD) decreases in MiaPaCa-2 cells but neither Hes-1, the nuclear target of NICD, nor the ratio Bax/Bcl-2 were affected. We further showed that in MiaPaCa-2 cells SELN6.0 induced the activation of NF-*k*B, which promotes the expression and the secretion of SDF-1α. This chemokine interacts with its receptor CXCR4 on MiaPaCa-2 cells and activates the Akt survival pathway protecting cells from death. This activation process promoted by exosomal lipids could have implications in tumor progression and drug resistance.

## INTRODUCTION

Exosomes are small vesicles released by many cell types [1], the biological significance of which is largely questioned [2]. Because of the abundance of signaling proteins and adhesion molecules at their surface [3], it is hypothesized that exosomes may serve as vehicles for long-range intercellular communications [4, 5]. Upon interaction with their target cells, exosomes deliver their information in a lipid-forming microdomains (termed thereafter raft) dependent mechanism [6]. The transfer of proteins or of genetic material and the subsequent biological effects on cell fate are well described [1, 3, 4]. However impacts of exosomal lipids, which are also transferred [7], on cell behavior remain largely ignored. We recently reported that the human pancreatic cancer SOJ-6 cells expressed exosomal nanoparticles, rich in lipid-forming rafts [8]. These exosomal particles interacted with SOJ-6 cells to disturb the functioning of partners of the Notch-1 survival pathway localized in membrane lipid microdomains. In particular the γ-secretase complex, which cleaves Notch-1 in the active Notch IntraCytoplasmic Domain (NICD), is sensitive to its lipid microenvironment [9]. These effects on Notch functioning down-regulate the phosphorylation of the pro-apoptotic PTEN and GSK-3β, leading to their activation. Lastly, GSK-3β inhibits mitochondrial pyruvate dehydrogenase, up-regulates pro-apoptotic Bax protein, down-regulates anti-apoptotic Bcl-2, Hes-1 (a nuclear target of the Notch pathway) and cyclin D1 expressions, and promotes arrest of the cell cycle in the G_0_-G_1_ transition phase. We therefore demonstrated that the interaction of exosomes with tumor cells conspicuously involves Notch signaling to drive target cells (in part SOJ-6 pancreatic cells) towards apoptosis *via* the intrinsic pathway [10].

Based on three observations: 1/lipids seem to be required for the exosome capture by cells [8, 11], 2/proteins are not involved in the observed effects of exosomes on SOJ-6 pancreatic tumor cells proliferation inhibition [10] and 3/the lipid composition of cell death-inducing exosomes expressed by SOJ-6 cells are enriched in lipids forming raft domains, we yielded Synthetic Exosome-Like Nanoparticles (SELN) with similar lipid composition but lacking proteins. We designed two types of SELN with ratios of lipid ordered phase over lipid disordered phase equal to 3.0 or 6.0 [12]. As already described with exosomes [10] SELN were able to trigger inhibition of tumor SOJ-6 cells survival through the mitochondria-dependent cell apoptotic pathway [12]. In SOJ-6 cells, SELN rich in lipid-forming rafts (i.e SELN6.0) down-regulated the phosphorylation of pro-apoptotic PTEN and GSK-3β, leading to their activation. These SELN also decreased the expression of anti-apoptotic Bcl-2, meanwhile increasing that of pro-apoptotic Bax proteins. Furthermore SELN6.0 decreased the amount of NICD, which consecutively decreased the expression of Hes-1, its nuclear target. Although SELN affected the survival of human pancreatic cancer SOJ-6 cells *via* the Notch pathway inhibition, the MiaPaCa-2 cells were particularly resistant to exosomal particles and to SELN hypothetically due to the fact that this cell line poorly expresses Notch pathway partners [10, 12]. MiaPaCa-2 cells are also resistant to gemcitabine the gold-standard drug for pancreatic cancer therapies. This intrinsic resistance of MiaPaCa-2 cells to curative drugs has been attributed to their cancer stem-like cells or initiating cells characteristics, notably the aldehyde dehydrogenase (ALDH) overexpression [13, 14]. In pancreatic cancer this ALDH-expressing cell population is particularly sensitive to cyclopamine, an inhibitor of the Hedgehog self-renewal embryonic pathway [15], one of the numerous misregulated signaling pathways in pancreatic cancer [16]. We wondered whether the resistance of MiaPaCa-2 cells to SELN6.0 could be either due to a time-delayed answer to SELN6.0 or to an antagonistic effect of these lipid particles on the inhibition of the Notch-1 survival pathway. The CXCR4-SDF-1α signaling axis has been implicated in pancreatic cancer drug resistance [17]. Therefore we hypothesized that the CXCR4-SDF-1α signaling axis could be involved in the resistance of MiaPaCa-2 cells.

Here we showed that in MiaPaCa-2 SELN-resistant cells [12] SELN6.0 impacted on the Notch-1 pathway as already observed with SELN-sensitive SOJ-6 cells but do not affect MiaPaCa-2 cells survival. We observed that SELN6.0 induced the activation of NF-*k*B transcription factor and its translocation towards the nucleus. We also demonstrated that SELN6.0 promote the expression and secretion of the SDF-1α chemokine. The interaction of SDF-1α with its dedicated receptor CXCR4 then activates the Akt pathway to ensure MiaPaCa-2 cells survival. Consequently the SDF-1α-CXCR4 axis is activated by SELN6.0 and counteracted their inhibitory effects on the Notch-1 pathway.

## RESULTS

### SELN6.0 impact on the Notch pathway but do not affect Hes-1 expression and the ratio Bax/Bcl-2

**Figure 1:**
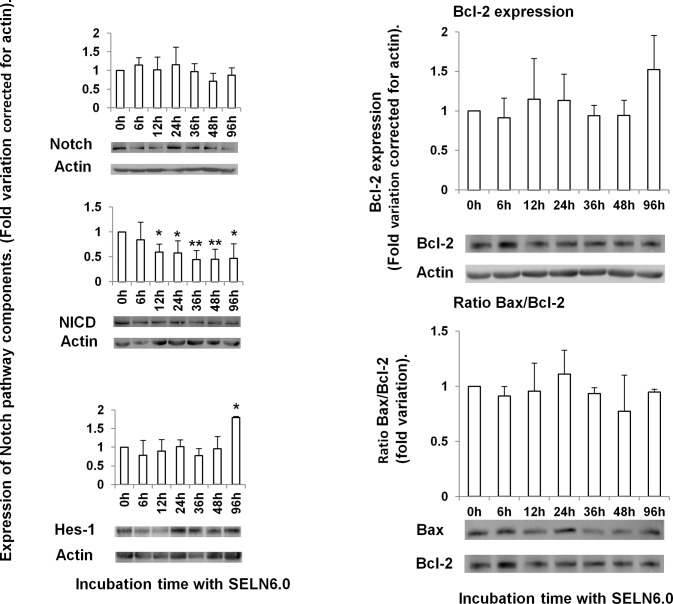
Effects of SELN6.0 on the Notch pathway in MiaPaCa-2 cells MiaPaCa-2 cells were grown until 60-70 % confluence and starved 24h prior incubation with SELN6.0 or PBS (control). At each time supernatants were removed, cells were lysed, centrifuged 30 min at 12000g to obtain proteins. 80 μg of proteins were loaded for electrophoresis and transferred onto a nitrocellulose membrane. After saturation, the membrane has been incubated overnight with the primary antibody as indicated, then washed and incubated with the required secondary POD antibody before detection. Variations were corrected for actin. (Mean +/− SD of 3 independent experiments).

We have previously shown that synthetic exosome-like nanoparticles composed of lipids and in which the ratio of total lipids forming ordered phase (Lo) over total lipids forming disordered phase, (Ld) has been fixed to 6 (referred to as SELN6.0) did not affect the MiaPaCa-2 cells survival after 24h incubation [12]. Therefore we wondered whether the survival inhibition promoted by SELN6.0 on MiaPaCa-2 cells due to Notch-1 pathway inhibition (as previously observed with SOJ-6 cells, [12]) could be time-delayed. For this purpose MiaPaCa-2 cells were incubated in the presence of SELN6.0 for time up to 96h. Only one dose of SELN6.0, corresponding to 16 nmoles cholesterol/ml has been used all along this study. This amount of cholesterol (determined using SELN6.0 labeled with [^3^H]-cholesterol, [12]) corresponds to that found in exosomes from SOJ-6 cells when used at 5 μg/ml in term of proteins allowing significant SOJ-6 cells survival inhibition [10]. As shown in Figure 1 (left panels), the expression of Notch-1 in MiaPaCa-2 cells is not affected by SELN6.0. In SOJ-6 human pancreatic tumoral cells the inhibition of Notch-1 by SELN6.0 implicated the decrease in γ-secretase activity to maturate Notch-1 in its active intracellular domain or NICD [12]. Therefore we have determined if the amount of NICD decreased upon MiaPaCa-2 cells incubation with SELN6.0. As shown in Figure 1 (left panels), the amount of NICD decreased with incubation time of MiaPaCa-2 cells with SELN6.0. This means that the γ-secretase involved in Notch-1 maturation is affected by SELN6.0 as already found in SOJ-6 cells [12]. However the expression of Hes-1, the cell nuclear target of NICD, is not affected in MiaPaCa-2 cells upon incubation with SELN6.0, it is even significantly increased after 96h incubation (Figure 1, left panels). Because the decrease in NICD drove SOJ-6 cells to apoptosis by increasing the ratio Bax over Bcl-2 [12] we next evaluated this ratio in MiaPaCa-2 cells upon incubation with SELN6.0 for time up to 96h. Figure 1 shows that neither the anti-apoptotic Bcl-2 protein expression (right upper panel) nor the ratio Bax over Bcl-2 (right lower panel) is affected with time upon incubation of MiaPaCa-2 cells with SELN6.0. This means that the effects of SELN6.0 on the Notch pathway are somehow antagonized. As a consequence MiaPaCa-2 cells survival cannot be affected by SELN6.0.

### SELN6.0 promote the activation of the nuclear transcription factor *k*B (NF-*k*B)

**Figure 2:**
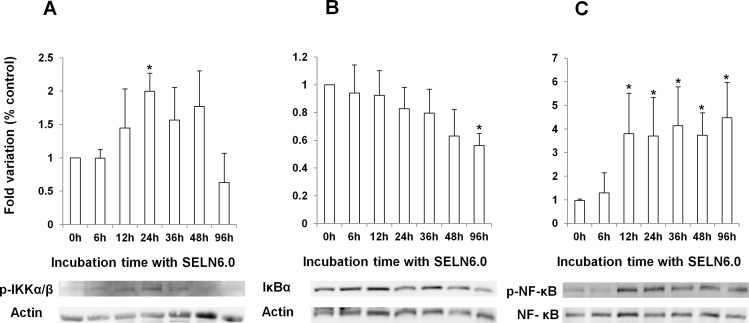
Effects of SELN6.0 on the NF-kB signaling MiaPaCa-2 cells were grown until 60-70% confluence and starved 24h prior incubation with SELN6.0 or PBS (control). At each time supernatants were removed, cells were lysed, centrifuged 30 min at 12 000g to obtain proteins. 80 μg of proteins were loaded for electrophoresis and transferred onto a nitrocellulose membrane. After saturation, the membrane has been incubated overnight with the primary antibody to Ser176/180-phosphorylated p-IKK (A) and to Ser32-phosphorylated I*k*B (B) and to Ser536 NF-*k*B (C) as indicated, then washed and incubated with the required secondary POD antibody before detection. Values were corrected for actin (A, B) or for total NF-*k*B (C). (Mean +/− SD of 3 independent experiments).

**Figure 3:**
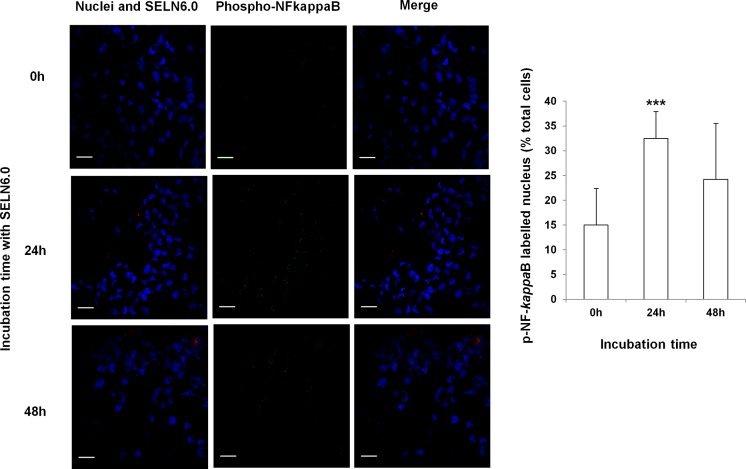
Effects of SELN6.0 on the phosphorylated NF-*k*B nuclear translocation MiaPaCa-2 cells were seeded on 1.2 cm-diameter cover slips in 12-well plate, once adherent cells were incubated with *N*-Rh-PE labelled SELN6.0 for 0, 24 and 48h (red dots). At the end of the incubation time cells were washed with PBS and then fixed. Cells were permeabilized with 0.1 % saponine-PBS during 30 min at room temperature, then cells were saturated (4% BSA 0,1% saponine in PBS) and were incubated with primary antibody to phosphorylated NF-*k*B, during 90 min. After washes cells were incubated with Rabbit Alexa Fluor 488 secondary antibody (green dots) and nuclei were blue-colored with Draq5 at room temperature for 30 min. (Scale bar = 500 μm). Graph represents the counting of 6 areas randomly selected from independent experiments. Between 300 and 450 total cells were counted and green dots containing nuclei were reported to total nuclei number. Data are mean +/− SD.

Given the cross-talk between Notch-1 and the canonical NF-*k*B pathways [18, 19] we hypothesized that the latter pathway could be involved in the MiaPaCa-2 cells survival observed upon SELN6.0 exposure. As shown on Figure 2A, I*KK* kinase (IKKα/β) phosphorylation at residues Ser176/Ser180 increased after 12h incubation of MiaPaCa-2 cells with SELN6.0 to reach a significant difference after 24h incubation. The phosphorylation then decreased to the basal level after 96h incubation. Meanwhile the expression of the NF-*k*B inhibitor, I*k*Bα, decreased with SELN6.0 incubation time of MiaPaCa-2 cells to reach significant decrease at 96h incubation (Figure 2B). I*k*Bα phosphorylation at Ser32 also decreased with time while the ratio Ser32phosphorylated-I*k*Bα over total I*k*Bα is constant and closed to the unit with incubation time (data not shown) meaning that I*k*Bα once phosphorylated is rapidly degraded [20]. As a consequence NF-*k*B (RelA, p65) is phosphorylated (*i.e.* activated, [20]) on Ser536 (Figure 2C) and translocated to the nucleus (Figure 3). These data suggested that SELN6.0 induced the activation of the NF-*k*B *via* p65 phosphorylation with a significant activation observed after 12h incubation time.

### SDF-1α is involved in MiaPaCa-2 cells survival

**Figure 4:**
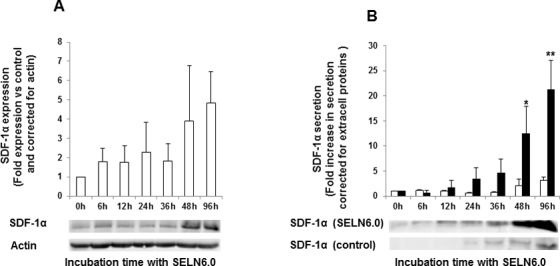
Expression of Stromal Derived Factor (SDF-1α) by MiaPaCa-2 cells A : MiaPaCa-2 cells were grown until 60-70 % confluence and starved 24h prior incubation with SELN6.0 or PBS (control). At each time supernatants were removed, cells were lysed, centrifuged 30 min at 12 000g to obtain proteins. 80 μg of proteins were loaded for electrophoresis and transferred onto a nitrocellulose membrane. After saturation, the membrane has been incubated overnight with the primary antibody to SDF-1α then washed and incubated with the secondary POD antibody before detection and variations were corrected for control and reported to actin. (Mean +/− SD of 4 independent experiments). B : Secretion of SDF-1α in MiaPaCa-2 cells conditioned medium. MiaPaCa-2 cells were grown until 60% confluence and starved 24h prior incubation with SELN6.0 (full column) or PBS only (control, empty column). At indicated time supernatants were removed, dialyzed against water, lyophilized, dissolved back in 1.6 ml distilled water and precipitated. The pellet was taken back in water, diluted in TS/TD and 35 μl were loaded for electrophoresis and transferred onto a nitrocellulose membrane. After saturation, the membrane has been incubated overnight with antibodies to SDF-1α then washed and incubated with the secondary POD antibody before detection and variations were corrected for extracellular protein concentration. (Mean +/− SD of 3 independent experiments).

**Figure 5:**
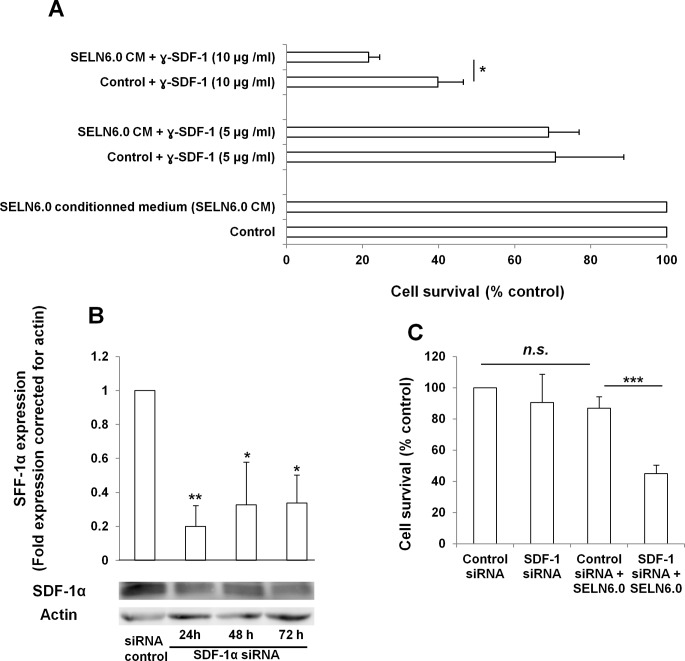
SDF-1α is involved in MiaPaCa-2 cells resistance to SELN6.0 A : Effect of SDF-1α blocking antibody. MiaPaCa-2 cells were grown until 60% confluence and starved 24h prior incubation with SELN6.0 or PBS (control) during 48h. Conditioned medium were removed, dialyzed against water (overnight, 4°C, Cut-off 1 kDa) and lyophilized. Lyophilized conditioned medium were taken back into fresh medium, filtered on 0.2 μm filters and used for the culture of naïve MiaPaCa-2 cells (grown in 96-wells plates at 6 000 cells by well) in the presence of 0, 5 and 10 μg/ml SDF-1α blocking antibodies for 24h. (Mean +/− SD of 3 independent experiments based on at least 8 points measurements). B : Effect of SDF-1α specific siRNA on SDF-1α expression. MiaPaCa-2 cells were plated in 6-well tissue culture plates at a density of 3 × 10^5^ cells / well. Prior transfection, the culture medium was removed and replaced with OPTI-MEM culture medium. Cells were transfected either with the mix of SDF-1α siRNA or the control siRNA at a concentration of 100 nM. Cells were then lysed and proteins loaded for electrophoresis and transferred onto a nitrocellulose membrane. After saturation, the membrane has been incubated overnight with primary antibody to SDF-1α then washed and incubated with the secondary POD antibody before revelation. (Mean +/− SD of 3 independent experiments). C : Effect of SDF-1α specific siRNA mix on MiaPaCa-2 cells in the presence of SELN6.0. 24 h after transfection with SDF-1α or control siRNA, MiaPaCa-2 cells were seeded in 96 well plates (6 000 cells/well) as soon as cells became adherent they were starved one night. Then, 48 hours after transfection, cells were challenged with SELN6.0 during 24 hours (total time 72h after transfection). Finally cell survival has been assessed through a MTT test. (Mean +/− SD of 3 independent experiments based on at least 8 points measurements each).

The transcription factor NF-*k*B plays a key role in the control of gene expression accounting for the up-regulation of cytokines and chemokines [20, 21]. The α-chemokine SDF-1α has been implicated in pancreatic cancer cells resistance to drugs used in therapy [17]. Also a subpopulation of CXCR4 (a receptor for SDF-1α) positive pancreatic cancer cells has been implicated in tumor growth [22]. Finally recombinant SDF-1α has been shown to reverse the SOJ-6 cell survival inhibition promoted by SELN6.0 (S. Beloribi-Djefaflia, unpublished results). Therefore we sought to determine whether SDF-1α may be involved in the resistance of MiaPaCa-2 cells to SELN6.0. Following MiaPaCa-2 cells incubation with SELN6.0 we observed the expression of SDF-1α. Although intracellular expression of SDF-1α increased with time (Figure 4A) this increase does not reach significance likely due to inter-experiment variations. Further and after a necessary concentration as pancreatic cells did not express large amount of the CXCR4 ligand [23], SDF-1α can be detected in the conditioned medium. This extracellular expression became significant after 48h incubation with SELN6.0 (Figure 4B). Furthermore the ratio of SDF-1α expressed by MiaPaCa-2 cells versus that secreted in the cell culture medium suggested that SDF-1α expression increased in MiaPaCa-2 cells after only 6-12h incubation with SELN6.0. This means that the increase in the SDF-1α expression precedes its secretion in the conditioned medium (Supplementary Figure 1A). To confirm that SDF-1α is actually involved in MiaPaCa-2 cells survival upon SELN6.0 incubation, a blocking antibody to SDF-1α has been used. As shown in Figure 4B the amount of SDF-1α present in MiaPaCa-2 cells conditioned medium is significantly increased upon a 48h-96h incubation of cells with SELN6.0. Therefore MiaPaCa-2 cells were incubated for 48h in the presence or in the absence of SELN6.0. Conditioned medium (10 ml) were then withdrawn and dialyzed overnight against water, lyophilized, then taken back in fresh medium and filtrated before using as medium to grow naïve MiaPaCa-2 cells. As shown on Figure 5A, the blocking antibody to SDF-1α used at 5 μg/ml had no effect on MiaPaCa-2 cells survival whatever the cell incubation with control conditioned medium or with SELN6.0 conditioned medium. However increasing the concentration of the blocking antibody up to 10 μg/ml significantly decreased the MiaPaCa-2 cells survival of both cells incubated with control-conditioned medium (a still possible off-target effects of antibodies on cells) or with SELN6.0-conditioned medium, but the decrease in cells survival is significantly higher for naïve MiaPaCa-2 cells cultured in the presence of SELN6.0-conditioned medium than in those grown in control-conditioned medium. We further invalidated the expression of SDF-1α in MiaPaCa-2 cells using siRNA. For this purpose MiaPaCa-2 cells were transfected with a mix of SDF-1α silencing specific siRNA (100 nM) to avoid off-target effect or with a scrambled (control, 100 nM) siRNA. The SDF-1α knock-down is observed at least during 72 hours following transfection (Figure 5B). Therefore once transfected, cells were incubated for 24h with or without SELN6.0 before measuring cell survival. As shown in Figure 5C, following SDF-1α silencing by specific siRNA, the MiaPaCa-2 cells survival does not vary when compared to the control siRNA transfected cells. However when MiaPaCa-2 cells following SDF-1α silencing were incubated with SELN6.0 for 24 h, the cell survival is significantly decreased when compared to control siRNA transfected cells. Also the survival of MiaPaCa-2 cells following SDF-1α silencing and incubated with SELN6.0 for 24h is significantly decreased when compared to the survival of these SDF-1α siRNA transfected cells without incubation with SELN6.0.

### Conditioned medium of SELN6.0-treated MiaPaCa-2 cells induces cell resistance

**Figure 6:**
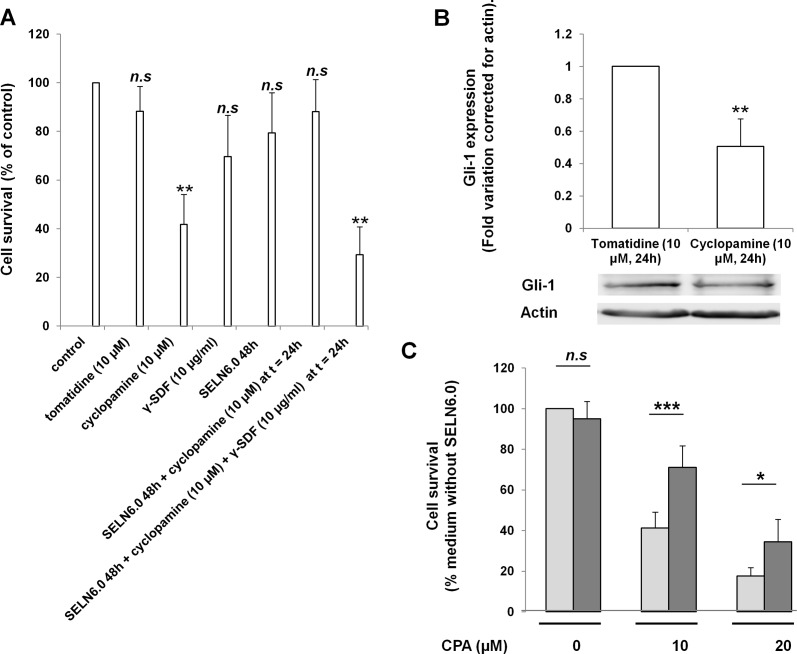
Effects of cyclopamine, SELN6.0 and conditioned medium on MiaPaCa-2 cells survival A: MiaPaCa-2 cells were seeded in 96 wells plates (4000 cells/well), then starved during 24h. Cells were incubated with tomatidine, cyclopamine, blocking antibodies to SDF-1α (γ-SDF) during 24h or with SELN6.0 during 48h then cyclopamine was added at t =24h with or without γ-SDF for an extra 24h incubation. At the end cell survival was assessed by MTT test. (Mean +/− SD of 3 independent experiments). B: MiaPaCa-2 cells were grown until 60-70 % confluence and starved 24h prior incubation with tomatidine or cyclopamine for 24h. At the end of the incubation time, cells were lysed, centrifuged 30 min at 12 000g to pellet cell debris. Then 80 μg of proteins present in supernatant were charged for electrophoresis and transferred onto a nitrocellulose membrane. After saturation, the membrane has been incubated with Gli-1 primary antibody overnight, then washed and incubated with the required secondary HRP antibody before detection and variations were reported to actin. (Mean +/− SD of 3 independent experiments). C: MiaPaCa-2 cells were grown in 10 cm diameter dishes until 60-70 % confluence before starvation for 24h. Then they were incubated with freshly prepared SELN6.0 (black column) or with PBS (grey column) during 48h. Media were collected, dialyzed overnight against water at 4°C and lyophilized. In parallel naïve MiaPaCa-2 cells were grown in 96 wells plates (6 000 cells/well) and starved for 24h. Then lyophilized media were diluted with fresh medium, filtered on 0.2 μm filters and used for the culture in 96 wells plates with or without cyclopamine (CPA, 0 μM, 10 μM and 20 μM). After 24h, cell survival was assessed by a MTT test. (Mean +/− SD of 3 independent experiments based on at least 12 point measurements each).

To go further in deciphering the mechanism by which SELN6.0 reverse the inhibition of the Notch-1 signaling pathway (Figure 1), a drug susceptible to inhibit the MiaPaCa-2 cells survival was required. MiaPaCa-2 cells are resistant to gemcitabine, thus in an attempt to find out this drug we examined the statute of this cell line. Flow cytometric analyses indicated that 79.5 +/− 11.4 % of MiaPaCa-2 cells were ALDH positive, an accepted common feature of cancer stem cells [11]. Further 85.3 +/− 10.4 % of these cells also expressed CD44 (a cancer stem cell related surface marker, [24]). MiaPaCa-2 cells were also susceptible to form primary and secondary pancreatospheres (data not shown) meaning that MiaPaCa-2 cells display some phenotype characteristics of stem-like cancer cells or initiating cancer cells. MiaPaCa-2 cells also express pluripotency maintaining transcription factors such as Nanog and Oct4 (data not shown, [25]). Those data confirm the cancer stem-like or cancer initiating statute of the MiaPaCa-2 cell line [13, 14]. Therefore we turn out to cyclopamine (CPA), an antagonist of the Hedgehog self-renewal embryonic pathway which is significantly expressed in cancer promoting cells. CPA is reported to induce apoptosis in pancreatic tumor cells [26] and to reduce the percentage of cells expressing the pancreatic cancer stem cell marker ALDH [15]. As expected CPA significantly inhibits MiaPaCa-2 cells survival, contrary to its structurally analog tomatidine (Figure 6A). This effect is reversed by a pre-incubation of cells with SELN6.0 before adding CPA. The trapping of CPA by SELN6.0 may not account for the reversed effect as the IC_50_ for MiaPaCa-2 survival inhibition by CPA is not affected by SELN6.0 (Supplementary Figure 2). We then verified that CPA affected the Hedgehog signaling pathway taking the cell expression of Gli-1 as a read-out of the drug effect. As shown in Figure 6B the expression of Gli-1 (160 kDa) was significantly decreased by CPA used at the IC_50_ (Supplementary Figure 2) for 24h. Further MiaPaCa-2 cells were incubated in the presence or in the absence of SELN6.0 and control- and SELN6.0-conditioned medium were used to grow naïve MiaPaCa-2 cell (see above) before adding CPA at the final concentration of 0, 10 and 20 μM. As shown on Figure 6C, MiaPaCa-2 cells survival was not affected by SELN6.0-conditioned medium compared to control-conditioned medium. However and independently of CPA concentration, the CPA effect on cell survival was less pronounced when SELN6.0-conditioned medium was used as cell culture medium instead of the control conditioned medium. As also shown in Figure 6A blocking antibodies to SDF-1α reverse the protective effect of SELN6.0 on CPA inhibition. This result strongly suggested that SDF-1α present in the SELN6.0 conditioned medium protect from or reverse the inhibitory effect of CPA on MiaPaCa-2 cells survival. Therefore CPA was further used as a tool in following experiments to record the reversion of SELN6.0 protective effects on MiaPaCa-2 cells.

### The SDF-1α-CXCR4 axis is responsible for MiaPaCa-2 cell resistance

**Figure 7:**
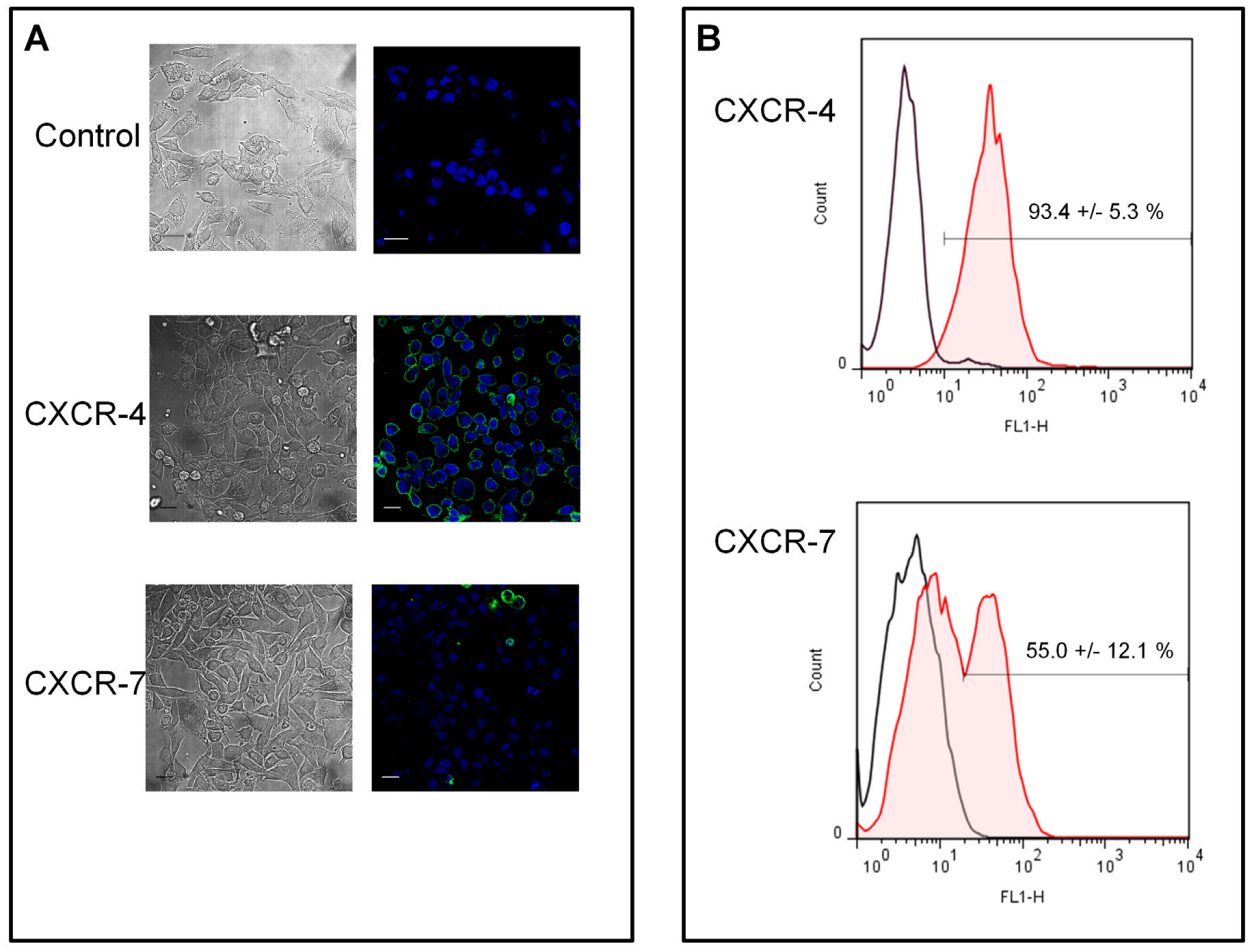
Expression of CXCR4 and CXCR7 by MiaPaCa-2 cells A : MiaPaCa-2 cells were seeded on 1.2 cm-diameter cover slips in 12-wells plate, once adherent cells were seeded in appropriate medium on cover-slips in 12 well-plates. Cells were fixed (2 % paraformaldehydein PBS, 37 °C, 15 min) and saturated (4% BSA in PBS, 30 min). The cells were then incubated successively with the primary antibodies to CXCR4 or to CXCR7 for 90 min and then with secondary antibody to IgG coupled to AlexaFluor 488 for 45 min. The cell nuclei were labelled 30 min with 1 μM Draq5, a far-red fluorescent DNA dye. All the later stages were carried out at 4°C. (Scale bar = 500 μm). B: Subconfluent monolayers of MiaPaCa-2 cells were harvested and suspended in DMEM containing 10 % FCS during 30 min at 37 °C. The single cell suspension (10^6^ cells/ml) was incubated for 90 min at 4°C in the presence of antibodies to CXCR4 or to CXCR7. Cells were rinsed three times with ice-cold PBS and then incubated for 45 min at 4°C with the appropriate secondary conjugated antibody. Cell-bound fluorescence was quantified (Flowjo program). Each value represents the mean fluorescence per cell. Non-specific labeling was determined by incubating cells with the secondary antibody alone (control). Graphs are representative of tree independent experiments and data represent the mean +/− SD obtained from these separate experiments.

**Figure 8:**
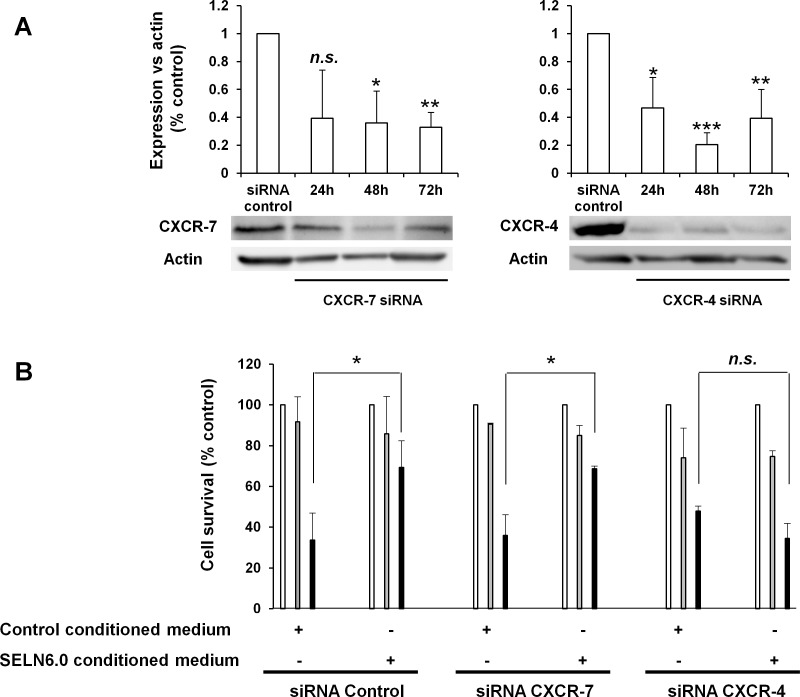
CXCR4 is in involved in MiaPaCa-2 cells resistance to SELN6.0 A : Effect of CXCR7 and CXCR4 specific siRNA on respective expression. MiaPaCa-2 cells were plated in 6-well tissue culture plates at a density of 3 × 10^5^ cells / well. Prior transfection, the culture medium was removed and replaced with OPTI-MEM culture medium. Cells were transfected either with the mix of CXCR7 or CXCR4 specific siRNA or the control siRNA at a concentration of 100-150 nM. Cells were then lysed and proteins loaded for electrophoresis and transferred onto a nitrocellulose membrane. After saturation, the membrane has been incubated overnight with primary antibody to CXCR4 and CXCR7 then washed and incubated with the secondary POD antibody before revelation. (Mean +/− SD of 3 independent experiments). B : Effect of CXCR4 and CXCR7 specific siRNA on MiaPaCa-2 cells in the presence of SELN6.0. 24 h after transfection with CXCR4 and CXCR7 specific siRNA or control siRNA, MiaPaCa-2 were seeded in 96 well plates (6 000 cells/well) as soon as cells became adherent they were starved one night. Then, 48 hours after transfection, cells were cultured in control or SELN6.0 conditioned medium (see Figure 8A) and challenged with SELN6.0 or with CPA (10 μM) during 24 hours (total time 72h after transfection). Finally cell survival has been assessed through a MTT test. (Mean +/− SD of 3 independent experiments based on at least 8 points measurements each). White column; cell survival in the presence (+) or absence (−) of conditioned medium of control cells or of SELN6.0 pre-incubated cells. Grey and black columns; cell survival in the presence (+) or absence (−) of conditioned mediums of control cells or of SELN6.0 pre-incubated cells with added SELN6.0 (grey) or with added CPA (10 μM, black).

In the next step we attempted to determine which of the SDF-1α receptor, CXCR4 or CXCR7 was the chemokine target. Virtually no pancreatic cancer cell possessed dual cell surface expression of SDF-1α receptors i.e. CXCR4 and CXCR7 [27]. However and because this result is conflicting [28, 29] we firstly determined the expression of CXCR4 and of CXCR7 by MiaPaCa-2 cells. Flow cytometry experiments showed that 93.4 +/− 5.3 % of MiaPaCa-2 cells display a surface membrane expression of CXCR4 (Figure 7A, B). Although MiaPaCa-2 cells displayed a low reactivity on immuno-fluorescence experiment (Figure 7A), FACS analyses indicated that 55.0 +/− 12.1 % MiaPaCa-2 cells effectively do express the CXCR7 receptor (Figure 7B). Note that SELN6.0 do neither affect the surface expression of CXCR4 (data not shown) nor its expression level (Supplementary Figure 1B). We next invalidated CXCR7 and CXCR4 expression by mean of a mix of siRNA specific of each receptor. As shown in Figure 8A each mix respectively inhibit their target expression for time up to 72h, at least. Transfected cells were cultured in control-conditioned medium or in SELN6.0-conditioned medium (see above Figure 5), in the presence of SELN6.0 or 10 μM CPA. As shown in Figure 8B cells transfected with control siRNA were still insensitive to SELN6.0 and sensitive to CPA. However SELN6.0-conditioned medium still reverses the inhibition of cell survival promoted by CPA (left panel). The same profile was obtained with MiaPaCa-2 cells transfected with the siRNA targeting the CXCR7 expression meaning that the SDF-1α does not reverse the CPA effects *via* CXCR7 (central panel). Going further we showed that the invalidation of CXCR4 expression does not allow the reversion of the SELN6.0-conditioned medium effects on cell survival inhibition in the presence of CPA (right panel). This result demonstrates that CXCR4 is the target of SDF-1α. Taken as a whole those data demonstrated that 1/the CXCR4-SDF-1α axis seems inefficient in MiaPaCa-2 cells in normal conditions (*i.e.* in the absence of SELN6.0), and 2/this axis is activated in the presence of SELN6.0 to reverse the CPA effects on MiaPaCa-2 cells survival.

### SELN6.0 increase the Thr308 and Ser473 phosphorylation of Akt

**Figure 9:**
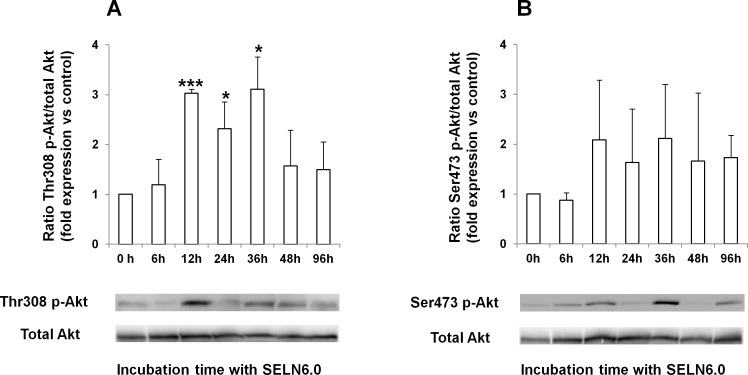
Phosphorylation of Akt in MiaPaCa-2 cells upon incubation with SELN6.0 MiaPaCa-2 cells were grown until 60-70 % confluence and starved 24h prior incubation with SELN6.0 or PBS (control). At each time supernatants were removed, cells were lysed, centrifuged 30 min at 12 000g to obtain proteins. 80 μg of proteins were loaded for electrophoresis and transferred onto a nitrocellulose membrane. After saturation, the membrane has been incubated overnight with the primary antibody to total Akt, to Thr308-phosphorylated Akt (Thr308 p-Akt, A) and to Ser473-phosphorylated Akt (Ser473 p-Akt, B) as indicated, then washed and incubated with the required secondary POD antibody before detection. Values were reported to total Akt. (Mean +/− SD of 3 independent experiments).

Given that 1/Akt is a downstream target of the CXCR4-SDF-1α axis [30] resulting in enhanced proliferation of pancreatic cancer cells [31] and 2 /that Akt has been associated with chemoresistance of pancreatic cancer [32] we have determined the Akt phosphorylation state in MiaPaCa-2 cells following incubation with SELN6.0 for time up to 96h. Akt can be phosphorylated on Thr308 and on Ser473 [33] albeit the phosphorylation of the latter residue is widely used as a marker for Akt activity, phosphorylation at residue Thr308 seems to promote a higher Akt activity [34, 35]. Although Ser473 and Thr308 can be independently phosphorylated [35] Ser473 phosphorylation can either facilitate Thr308 phosphorylation [36] or determine Akt substrates specificity [37]. Upon SELN6.0 incubation of MiaPaCa-2 cells Akt can be phosphorylated on both Thr308 and Ser473 (Figure 9). The phosphorylation of Thr308 becomes significant after 12h incubation then seems to decrease after 48h incubation. The Ser473 phosphorylation seems to parallel the Thr308 phosphorylation although values are less significant due to a delayed response observed in one experiment (Supplementary Figure 3).

## DISCUSSION

We have previously shown that exosome lipids are key-factors in the induction of human pancreatic cancer SOJ-6 cells death [8, 10]. This was latter confirmed by further experiments demonstrating that synthetic exosome-like nanoparticles or SELN were susceptible to evoke the SOJ-6 cells death *via* the inhibition of the Notch-1 survival pathway [12]. Further we also showed that the MiaPaCa-2 cells, another human pancreatic cancer cell line issued from an undifferentiated adenocarcinoma of the pancreas, was insensitive to SELN, and in part to SELN6.0 in whose the ratio raft lipids over phospholipids was fixed to 6.0. Although we have hypothesized that the resistance of MiaPaCa-2 cells to SELN6.0 was due to a low expression of the Notch-1 pathway partners [12] we wondered whether the MiaPaCa-2 cells resistance to SELN6.0 was due to a time-delayed response. Here we showed that even after 96h incubation SELN6.0 had no effect on MiaPaCa-2 cell survival. We next recorded that the generation of the intracellular domain of Notch (NICD) was decreased in MiaPaCa-2 cells upon incubation with SELN6.0. This result means, as already observed with SELN6.0-sensitive SOJ-6 cells, that SELN6.0 affected the Notch survival pathway and in part the γ-secretase complex involved in NICD release [12]. Also the ratio of the proapoptotic Bax protein versus the anti-apoptotic Bcl-2 protein is unaffected in MiaPaCa-2 cells upon incubation with SELN6.0. Consequently the effects of SELN6.0 on MiaPaCa-2 cells fate such as the inhibition of the Notch-1 survival pathway could be somehow antagonized by SELN6.0 themselves. The expression of Hes-1, one of the NICD target genes was not decreased by SELN6.0, opposite to what was observed with SOJ-6 cells [12]. A significant degradation of I*k*B can be observed in MiaPaCa-2 cells upon 96h incubation with SELN6.0 (Figure 2). One likely explanation is that I*k*B can be no more recruited to the *Hes1* promoter to repress its expression [38]. This explains the significant increase in Hes-1 expression upon 96h incubation of MiaPaCa-2 cells with SELN6.0 (Figure 1) and sustains the involvement of the NF-*k*B pathway in the MiaPaCa-2 cells resistance to SELN6.0. Therefore and given the link between Notch and NF-*k*B pathways [18, 19] we turn out to this last pathway. We thus tested whether NF-*k*B activation can be observed following MiaPaCa-2 cells incubation with SELN6.0. Here we observed the activation of the canonical NF-*k*B pathway with IKKα/β phosphorylation at Ser176/Ser180, degradation (following Ser32 phosphorylation) of I*k*Bα and Ser536 phosphorylation of the p65 subunit (RelA) of NF-*k*B along with the translocation of phosphorylated NF-*k*B towards the nucleus.

It is well established that NF-*k*B controls the gene expression of cytokines and chemokines accounting for increased expression [20, 21]. Consequently activation of NF-*k*B by SELN6.0 may account for SDF-1α secretion by MiaPaCa-2 cells upon incubation with SELN6.0 as observed here. In pancreatic cancer the CXCR4-SDF-1α survival axis [39] is associated with the pancreatic cancer drug resistance [17]. As shown here some 90% of MiaPaCa-2 cells expressed CXCR4 and to a lower extent CXCR7 α-chemokine receptors. The secretion of SDF-1α seems to be induced by SELN6.0 as earlier as 12h following MiaPaCa-2 cells incubation with lipid nanoparticles. However due to inter-experiment variations significant difference is reached for longer incubation time (Figure 4). Data mean that low SDF-1α concentrations may support MiaPaCa-2 cells survival. Thus SELN6.0 may prime MiaPaCa-2 cells to modulate the CXCR4 incorporation/conformation in rafts [40] allowing them to better respond to low SDF-1α concentrations [41]. Further, 1/a blocking antibody to SDF-1α annihilates the protective effect of the SELN6.0-conditioned medium on MiaPaCa-2 cells survival and 2 / the silencing of SDF-1α expression by a specific siRNA makes MiaPaCa-2 cells sensitive to SELN6.0. Although the SDF-1α-CXCR4 and SDF-1α-CXCR7 axes were each involved in cancer cell survival [28, 29] we showed here that 1 / CXCR4 is the main represented receptor for the α-chemokine SDF-1α on MiaPaCa-2 cell membranes. 2/the SDF-1α-CXCR4 signaling pathway seems to be preponderant over the SDF-1α-CXCR7 axis. Consequently SELN6.0 induce the expression and secretion of SDF-1α, which further bounds to the CXCR4 receptor to counteract the effect of SELN6.0 themselves on Notch pathway.

The Akt signaling may be the downstream survival pathway following the CXCR4/SDF-1α axis activation [30]. Therefore we determined the phosphorylation of Akt, the activation of which enhances pancreatic cancer proliferation [31] and drug resistance [32]. Here we showed that Akt is phosphorylated on both Thr308 and Ser473 that correlates with the highest activity of Akt [33]. The activation of Akt is significant after 12h incubation of MiaPaCa-2 cells with SELN6.0 then decreases after 48-96h incubation. It has been argued that oncogenic Ras activity acting as an upstream activator of Akt can stimulate the transcriptional activity of NF-*k*B (p65) [42]. Constitutive *K*ras and NF-*k*B activation are indentified as signatures of pancreatic ductal adenocarcinoma and RelAp65/NF-*k*B is constitutively activated in almost 70% of pancreatic cancer specimens and the inhibition of NF-*k*B activity inhibits pancreatic cell tumorigenesis [43, 44]. Therefore we have audited the status of our MiaPaCa-2 cell line for *K*ras mutation and determined that these cells harbor the *K*ras p-Gly12Cys homozygous mutation as already observed [45]. However the transactivation of NF-*k*B (p65) by Akt seems to be IKK-independent and independent of the liberation of NF-*k*B consecutive to the degradation of I*k*B [46, 47]. Furthermore this process could be restricted to PTEN-deficient cell models [46]. As shown here the IKK/I*k*B-dependent canonical NF-*k*B pathway is activated by SELN6.0. Also, PTEN is expressed and fully functional in MiaPaCa-2 cells [10]. In pancreatic cancer Akt may also activate NF-*k*B to induce the expression of Sonic Hedgehog, a ligand activating the Hedgehog embryonic survival pathway [39]. Such expression of Sonic Hedgehog was not observed upon MiaPaCa-2 cells incubation with SELN6.0 (data not shown). Taken altogether these data strongly suggest that in MiaPaCa-2 pancreatic cancer cells and in the presence of SELN6.0, Akt is likely a downstream target of the SDF-1α-CXCR4 axis rather than under *K*ras activity. Thus in MiaPaCa-2 SELN6.0-resistant cells SELN6.0 themselves evoke the SDF-1α expression and secretion which *via* the CXCR4 chemokine receptor activates the downstream Akt survival pathway [30]. Nevertheless the expression of mutated *K*ras in pancreatic cancer leads to the up-regulation of GSK-3 isoforms and to NF-*k*B activation [48]. As a consequence the constitutive activation of *K*ras in MiaPaCa-2 cells may be responsible for the basal activation of NF-*k*B [49, 50] as observed here. Furthermore MiaPaCa-2 cells are sensitive to the NF-*k*B inhibitor sulfasalazine [(IC50 = 1 mM approx, data not shown) and 51]. We have previously shown that fluorescent SELN6.0 following incubation with human pancreatic cancer cells colocalize with lipid rafts then fuse with the plasma membrane and diffuse/dilute within the plane of the membrane [12]. Once the fusion had occurred non-rafts lipids of SELN6.0 may freely diffuse within the cell membrane compartment to impact on the constitutively active oncogenic *K*ras which exhibits stable interaction with the inner leaflet of plasma membrane at saturable non-raft sites [52]. Such interaction is required for efficient signaling of GTP loaded Ras proteins [53]. Therefore the fusion of SELN6.0 with plasma membrane may affect the Ras location and facilitate signaling with the efficient activation of underneath proteins [53]. SELN6.0, impacting on *K*ras functioning, may lead to a significant over-expression of GSK-3 isoforms such as GSK-3β as we previously observed with MiaPaCa-2 cells upon SELN6.0 incubation [12], to the over-activation of NF-*k*B [49, 50], to its subsequent transcription activity on SDF-1α expression [20] and finally to the Akt survival pathway over-activation *via* the survival CXCR4-SDF-1α axis.

As mentioned in the introduction, MiaPaCa-2 cells are also resistant to gold-standard drugs, a phenotype attributed to the ALDH over-expression characterizing stem-like cancer or cancer initiating cells [14]. In pancreatic cancer the ALDH-expressing population is particularly sensitive to cyclopamine, an inhibitor of the Hedgehog self-renewal embryonic pathway [15, 16]. As shown here a large fraction of MiaPaCa-2 cells are ALDH positive and display some characters of stem-like cancer or cancer initiating cells such as the expression of the cancer stem-cell CD44 marker, of embryonic transcription factors Nanog and Oct4 [25]. These cells are also prone to generate primary and secondary pancreatospheres [54]. We further confirmed that MiaPaCa-2 cells were sensitive to the Hedgehog pathway inhibitor cyclopamine [55] which affects the expression of the Hedgehog intracellular target gene Gli-1. However the preincubation of MiaPaCa-2 cells with SELN6.0 impairs the cyclopamine effect and protects these cells against survival inhibition promoted by the Hedgehog antagonist. Blocking SDF-1α reversed the SELN6.0 protecting effects suggesting that SELN6.0 may also protect MiaPaCa-2 cells from drug deleterious effects.

In conclusion, this report shows that SELN6.0 induced the NF-*k*B activation and nuclear translocation in SELN6.0 insensitive human pancreatic tumoral MiaPaCa-2 cells. This nuclear factor likely promotes expression and secretion of the SDF-1α chemokine which following interaction with the CXCR4 cognate receptor may activate the Akt survival pathway thus protecting cells from death.

## MATERIALS AND METHODS

### Materials

Peroxidase (POD)-labelled goat antibodies to mouse IgG, FITC-labelled antibodies to mouse IgG and antibodies to actin were from Sigma (St Louis, MO). POD-labelled antibodies to rabbit immunoglobulins (IgG), antibodies to Bax, to Akt, to (Ser473) phospho-Akt, to (Thr308) phospho-Akt, and the NF-*k*B pathway sampler kit were from Cell Signaling (Beverly, MA). Antibodies to Notch-1 (extracellular domain), to NICD (Notch IntraCytoplasmic Domain), to Hes-1, to Gli-1, to CXCR4 and blocking antibodies to SDF-1α were from Abcam (Cambridge, UK). Antibodies to Bcl-2 came from Dako (Glostrup, Denmark). Antibodies to SDF1α were indistinctly from Abcam or Cell Signaling. PE-labeled-antibodies to CD44 came from BioLegend (San Diego, CA). Antibodies to CXCR7 for confocal, for western-blots and for FACS experiments came respectively from Sigma and from R&D systems (Minneapolis, MN). Alexa Fluor 488-labelled antibodies to mouse and to rabbit IgG were from Invitrogen (Illkirch, France) as well as Alexa Fluor 647 antibodies to mouse IgG. CXCR4, CXCR7 and SDF-1α specific siRNA mix and dedicated controls siRNA (scramble) were from Santa Cruz Biotechnology (Santa Cruz, CA). Aldefluor kit came from Stem Cell Technology (Vancouver, Canada). Sphingolipids, phospholipids and 1,2-dioleoyl-sn-glycero-3-phosphoethanol-amine-N-(lissamine-rhodamine sulfone) were purchased from Avanti Polar Lipids Inc (Alabaster, AL) and all other lipids (pure grade) were from Sigma-Aldrich (St Quentin-Fallavier, France). DMEM cell culture medium, penicillin, streptomycin and trypsin-EDTA were from InVitrogen (**Carlsbad, CA**). MiaPaCa-2 cell line used in this study came from the American Type Culture Collection (ATCC-CRL-1420, Rockville, MD). Cyclopamine was from Sigma, its uneffective structural analogue tomatidine was from Calbiochem.

### Preparation of conditioned medium

MiaPaCa-2 cells were grown to reach 60-70 % confluence and starved during 24h. Then they were incubated with medium (0.5% FCS) in the presence of SELN6.0 (SELN6.0 conditioned medium) or of the diluant of SELN6.0 (PBS, control conditioned medium). After 48h incubation conditioned medium were removed, dialyzed against pure water (tubing cut-off 1 kDa, Spectrum Laboratories, Broadwick CA), lyophilised and rediluted in fresh medium and filtrated (20 μm filter, Millipore, Molsheim, France). Conditioned mediums used for western blotting were lyophilized and proteins were precipitated. Indeed the lyophilisate has been diluted in 1.6 ml of cold water and 400 μl of TFA (Sigma). The mix was placed overnight at 4°C and centrifugated 30 min, 14 000g, 4°C. The supernatants were removed and the pellet was washed with cold acetone (1 ml) and centrifugated 30 min, 14 000g, 4°C. Finally the pellet has been dissolved in 120 μl of cold water, 20 μl were kept for protein quantification using the bicinconinic acid (μBCA) assay (Pierce, Rockford, IL). The remaining material was saved for SDS-PAGE and western-blotting analyses (see further).

### Cell growth and cell survival

MiaPaCa-2 cells originating from human pancreatic adenocarcinoma were seeded in DMEM medium with 10% fetal calf serum (FCS) at 4,000 to 8,000 cells / well unless otherwise stated in a 96-well culture plates. Cells were then deprived in FCS (0.5 % for 24 h) and these quiescent cells were further treated with increasing amounts of effectors, in the absence of FCS. Cell survival was assessed by 3-(4,5-dimethythiazol-2-yl)-2,5-diphenyl (MTT) assay. All determinations were compared to those of cell controls without added effectors and taken as 100 %. Results are given as mean ± SD.

### Synthetic exosome-like nanoparticles (SELN)

Synthetic Exosome-Like Nanoparticles or SELN were synthesized from lipid stock solutions as already described [12] and calibrated to the amount of exosomes used in previous studies [8, 10]. Only SELN in which the ratio of total lipids forming ordered phase (Lo) over total lipids forming disordered phase (Ld), has been fixed to 6 (referred to as SELN6.0) were used in this study. The median diameter determined by electron microscopy ranges between 55 nm to 100 nm and did not change with time [12], which agrees with the stability of natural exosomes [56]. Density and size of SELN correlate with those of cell-expressed exosomes [4].

### Fluorescent labelling of SELN

Fluorescent SELN were synthesized as above after incorporating 0.1-0.2 % (total lipid weight) of 1,2-dioleoyl-sn-glycero-3-phosphoethanolamine-N-(lissamine-rhodamine sulfone) (or *N*-Rh-PE). Fluorescence spectra were recorded on a LS45 spectrofluorometer (Perkin Elmer, Courtaboeuf, France).

### siRNA transfection

MiaPaCa-2 cells were plated in 6-well tissue culture plates at a density of 3 × 10^5^ cells / well. Prior transfection, the culture medium was removed and replaced with OPTI-MEM culture medium (Gibco, Carlsbad, CA). Cells were transfected either with the mix of SDF-1α siRNA to avoid off-target effect or the scrambled oligonucleotides (control siRNA) at a concentration of 100 nM, using Oligofectamin (Invitrogen) according to manufacturer's instructions. After 6h of incubation FCS was added to the media (10 % final) and 24 hours after transfection 6 000-8 000 cells/well were seeded in 96-wells culture plates, as soon as cells became adherent they were starved overnight. Then, 48 hours after transfection, cells were challenged with SELN6.0 during 24 hours. Following CXCR4 (100 nM) and CXCR7 (150 nM) specific siRNA transfection MiaPaCa-2 cells were further cultured in the presence of control conditioned medium or of SELN6.0 conditioned medium for 24h and challenged with SELN6.0 or with CPA (10 μM) during 24 hours. Finally cell survival has been assessed through a MTT test (total time experiment : 72h). At each step a cell aliquot was taken and lysed for western blotting analyses (see further figure 8).

### Flow cytometry

Cells were grown to reach 80 % confluence, they were trypsinised following three washes with PBS and resuspended in 10% FCS medium and placed 30 min at 37°C, an aliquot was used for cells counting. After a centrifugation (3 min, 1 500g), cells were rinsed and centrifugated (3 min, 1500g) three times and saturated with 4 % bovine serum albumin in phosphate buffered saline (BSA-PBS) at 4°C during 30 min. Then 1.5.10^5^ cells were seeded in 96 wells plates to be incubated at 4°C during 90 min with the appropriate primary antibodies diluted in 1% BSA-PBS according to manufacturer's protocol. After three washes with cold PBS separated by centrifugations (5 min, 2000g), the secondary labelled antibody was added (1/400 in BSA-PBS) for 45 min. The secondary labelling step was not conducted when PE-labelled primary antibody to CD44 was used in the first step. Finally cells were washed after centrifugation (5 min, 2 000g) three times with cold PBS and resuspended in cold PBS, maintained at 4°C till the FACS analyses (Becton-Dickinson FACScan flow cytometer, Cellquest software). Results were interpreted thanks to the Flowjo program (http://www.flowjo.com/home/windows.html).

### Confocal microscopy

Cells were seeded in appropriate medium on cover-slips in 12 well-plates (BD Falcon, Le Pont-de-Claix, France). Once adherent cells were fixed (paraformaldehyde, 2 % in PBS, 37 °C, 15 min) and saturated (BSA, 4% in PBS, 30 min). The cells were then incubated successively with the primary antibodies to CXCR4 or to CXCR7 for 90 min, washed trice in fresh PBS and finally with the appropriate secondary antibody to IgG coupled to Alexa Fluor 488 for 45 min. The cell nuclei were labelled 30 min with 1 μM Draq5, a far-red fluorescent DNA dye (Biostatus Limited, Shepshed, UK). All the later stages were carried out at 4°C. For phospho-NF-*k*B analyses, adherent cells were starved during 24h before incubated with SELN6.0 for 0h, 24h or 48h, then cells were washed and fixed (paraformaldehyde, 2 % in PBS, 37 °C, 15 min). Membranes were permeabilized with PBS-0.1% saponine during 30 min at room temperature (RT). Then cells were saturated with 4 % BSA-0.1% saponine-PBS (30 min, RT), they were further incubated with primary antibody to phospho-NF-*k*B at RT during 90 min. After three washes, cells were incubated with the secondary Alexa Fluor488 antibody during 45 min at RT. Finally nuclei were labelled with Draq5 at RT during 30 min. For observations, Confocal Laser Scanning Microscopy experiments were performed using a Leica SP5 microscope coupled with a Leica scanning device (Leica Microsystems, Mannheim, Germany). Images were recorded with LAS AF Lite acquisition software and were analysed with the public-domain ImageJ software (NIH; http://rsb.info.nih.gov/nih-image/).

### SDS-PAGE and Western blottings

After treatment, the cells were washed three times with ice-cold PBS, harvested and pelleted by centrifugation. Pellets were washed twice and lysed at 4°C in 0.5 ml of lysis buffer (Tris 50mM, NaCl 200mM, MgCl2 2,5mM, Glycerol 10 %, NP-40 1%), protease inhibitors (Complete TM, Roche Diagnostics, Meylan, France) and phosphatase inhibitors cocktail (Sigma). After lysis, homogenates were vortexed (10 sec, three times) clarified by centrifugation at 12, 000 x g for 30 min at 4°C. An aliquot was saved for protein determination (μBCA assay). Proteins in reducing sodium dodecylsulfate (SDS) buffer were separated on 10 % polyacrylamide gels and 0.1% SDS. After electrophoretic migration, proteins were transferred onto nitrocellulose membranes and processed for immunoblotting by using appropriate primary and secondary POD-labelled secondary antibodies. After washes, membranes were developed with a chemoluminescent substrate [8] visualized using a G:BOX-iChemi (SynGene) gel imaging device as described by the manufacturer and analyzed with the NIH ImageJ program for band quantification. Each blot was stripped and probed with the α-actin antibodies to correct for gel loading. For phosphorylated proteins membranes were first probed with the antibodies to phosphorylated residues then membrane were stripped (TFA 5%, 15 min at room temperature) and further probed with the total protein antibodies. The ratio phosphorylated residue over total Akt or NF-*k*B was then determined. All western blot quantifications were done using the NIH ImageJ software. Western blots were repeated at least three times from independent experiment and one representative image is shown.

### Statistical analysis

Each experiment was done at least three times and results expressed as means ± SD. Difference between experimental groups were analyzed with the bio-stat TGV student test (http://marne.u707.jussieu.fr/biostatgv/?module=tests/student). Significance was set as (*) *P* ≤ 0.05; (**) *P*<0.01 and (***) *P*<0.001. *n.s.* stands for not significant difference.

## SUPPLEMENTARY MATERIAL FIGURES


