# Supplementary material for: Exosomal lipids induce human pancreatic tumoral MiaPaCa-2 cells resistance through the CXCR4-SDF-1α signaling axis
Source: Oncoscience. 2014 Nov 11;2(1):15–30. doi: 10.18632/oncoscience.96 (PMC4341461; doi:10.18632/oncoscience.96)
Supplement: Supplementary file 1 [file oncoscience-02-0015-s001.pdf]

Exosomal lipids induce human pancreatic tumoral MiaPaCa-2 cells resistance through the CXCR4-SDF-1α signaling axis

Supplementary Material

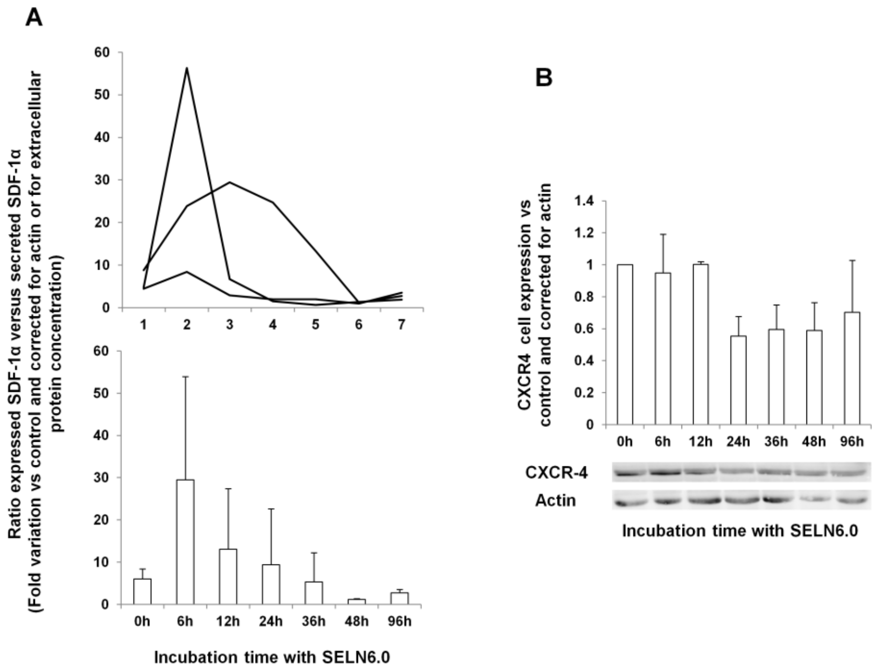

Supplementary Figure 1: Effects of SELN6.0 on the CXCR4-SDF-1α pathway. A : MiaPaCa-2 cells were grown until 60-70 % confluence and starved 24h prior incubation with SELN6.0 or PBS (control). At each time culture mediums were removed, cells were lysed, centrifuged 30 min at 12 000g to obtain proteins. 80 µg of proteins were loaded for electrophoresis and transferred onto a nitrocellulose membrane. After saturation, the membrane has been incubated overnight with the primary antibody to SDF-1α or to actin then washed and incubated with the secondary POD antibody before revelation. The amount of SDF-1α detected in each fraction was reported to actin and to control experiment. Removed culture mediums obtained in A were dialyzed against water (overnight, 4°C, Cut-off 1 000 Da) and precipitated. The pellet was taken back in water and 30 mg of protein were loaded for electrophoresis and transferred onto a nitrocellulose membrane. After saturation, the membrane has been incubated overnight with antibodies to SDF-1α then washed and incubated with the secondary POD antibody before revelation. The amount of secreted SDF-1α was reported to protein concentrations determined by µBCA assay in each culture medium. Then the ratio of expressed SDF-1α versus secreted SDF-1α was determined and plotted according to time of incubation with SELN6.0. Detail of the 3 independent experiments. **B**: MiaPaCa-2 cells were grown until 60-70 % confluence and starved 24h prior incubation with SELN6.0 or PBS (control). At each time supernatants were removed, cells were lyzed, centrifuged 30 min at 12 000g to obtain proteins. 80 µg of proteins were loaded for electrophoresis and transferred onto a nitrocellulose membrane. After saturation, membranes were been incubated overnight with the anti-CXCR4 primary antibody or with the actin antibodies then washed and incubated with the secondary POD antibody before revelation. Detail of the 3 independent experiments.

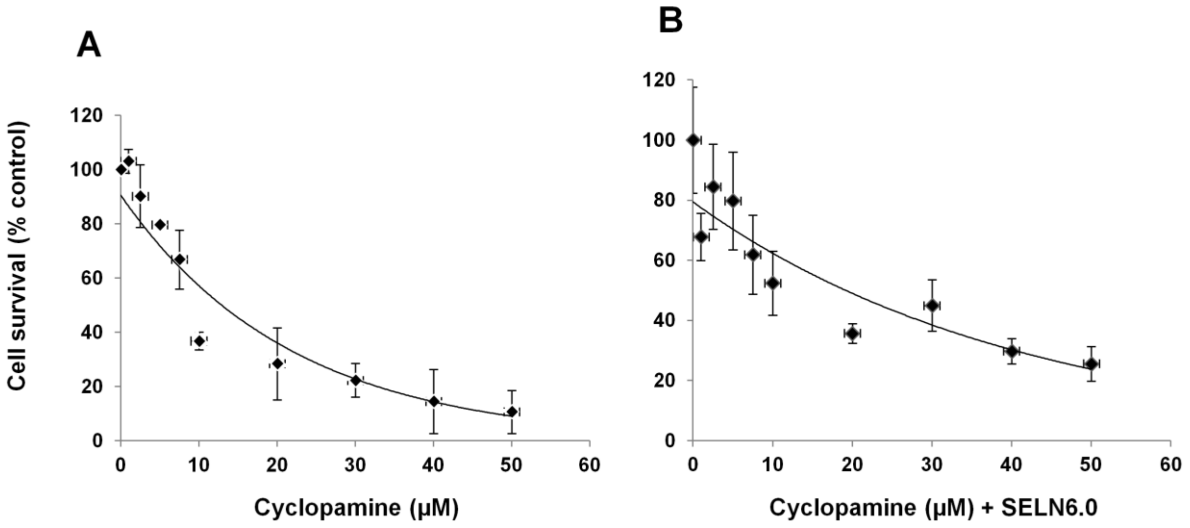

Supplementary Figure 2 : Effect of cyclopamine on MiaPaCa-2 cells survival. MiaPaCa-2 cells (6 000 cell/well in 96-wells plat were grown until 60%. Then cyclopamine alone (**A**) or cyclopamine concomitantly with SELN6.0 (**B**) was added to the culture medium at the indicated concentration. After 24h, cell survival was assessed by a MTT test. (Mean +/- SD of 3 independent experiments based on at least 12 point measurements each).

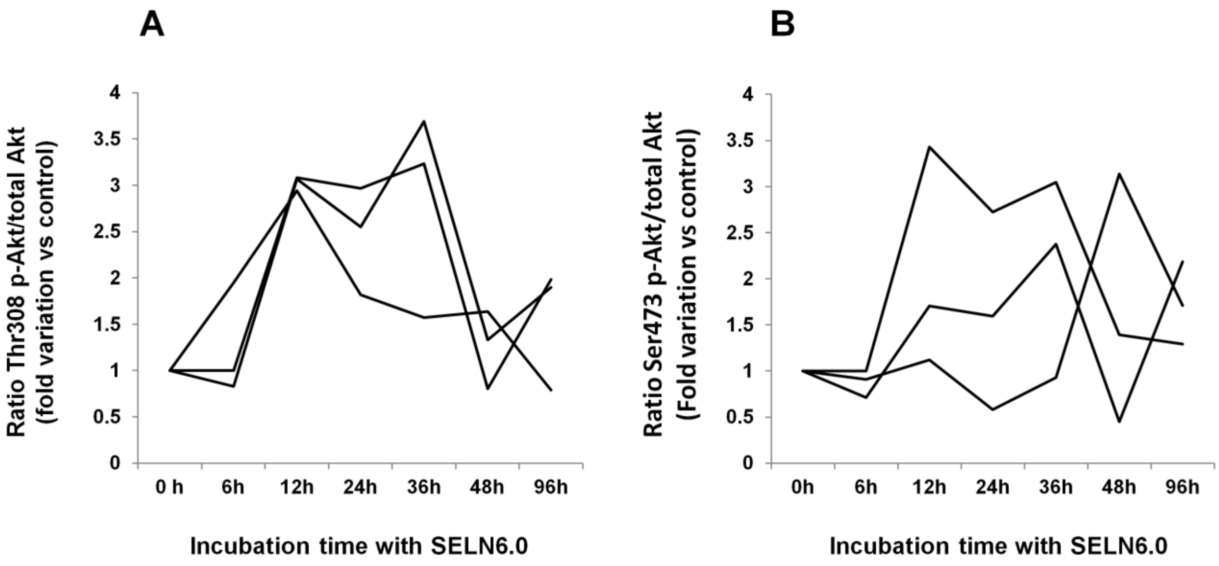

Supplementary Figure 3 : Phosphorylation of Akt in MiaPaCa-2 cells upon incubation with SELN6.0. MiaPaCa-2 cells were grown until 60-70 % confluence and starved 24h prior incubation with SELN6.0 or PBS (control). At each time supernatants were removed, cells were lyzed, centrifuged 30 min at 12000g to obtain proteins. 80 µg of proteins were loaded for electrophoresis and transferred onto a nitrocellulose membrane. After saturation, the membrane has been incubated overnight with the primary antibody to total Akt, to Thr308-phosphorylated Akt (Thr308 p-Akt, **A**) and to Ser473-phosphorylated Akt (Ser473 p-Akt, **B**) as indicated, then washed and incubated with the secondary HRP antibody before revelation. Detail of the 3 independent experiments.
